# The Differentiation of Abdominal Pain and Its Bearing on Treatment

**Published:** 1918-02

**Authors:** John B. Deaver

**Affiliations:** Philadelphia, Pa.


### THE DIFFERENTIATION OF ABDOMINAL PAIN AND ITS BEARING ON TREATMENT.

**By John B. Deaver, M.D., Sc.D., LL.D., Philadelphia, Pa.**

Pain has wisely been termed the guardian of health: the cry ot nature for assistance; a blessing in disguise for which we should be, but are not always, grateful, for no one "has as yet learned to love its rod." Its refining and ennobling influence on the disposition and character of erring mankind has been the theme of poet and novelist in all ages. Its significance to the internist and to the surgeon is self-evident, and I should have hesitated to adopt it for the subject of this evening's talk were it not that its language is oftentimes obscure and frequently misunderstood, and that what I have gained from a wide and varied experience might be of some use in reading aright some of its less clear indications, especially with reference to its location in the abdomen.

That there is a cause for every pain is a trite axiom, and our object should not be merely to relieve the pain but to remove the cause. Like many physical manifestations there is a psychological element in pain, so that it is difficult to estimate its severity. We cannot always rely on what our patients tell us as to its intensity or otherwise; what is excruciating to one may be passed off lightly by another. It is here that experience and that indefinable gift—intuition—are of so much value. Lacking these it is important that the clinician should disregard no complaint of pain. The subjective pains of the neurotic individual are not rarely found to be due to some pathological condition of the nervous sytem, while the more common objective pain is a distinct indication of tissue injury which dare not be ignored.

One of the greatest difficulties in the correct interpretation of pain is that the sensation is often reflected to regions remote from the actual site of the lesion causing it. This is perhaps better illustrated by pain in the abdomen, and also in the chest, than elsewhere in the body economy. It is a well-known fact that the abdominal organs are almost insensitive to pain, and that it is the exquisite sensitiveness of the abdominal wall that accounts for the prominence of pain in the symptom-complex of visceral disease. In other words, the stimulation of the nerve centers gives rise to pain which is referred to the peripheral distribution of these nerves in the body wall. Ordinarily we are not conscious of visceral functions. Peristalsis takes place and we know it not, but when for some reason or other it becomes violent, we are conscious of it in the form of pain, presumably due to disturbance of associated nerve tracts of the parties.

Abdominal pain is due to intrinsic and extrinsic causes. Not a few failures of diagnosis in supposed abdominal disease are due to lack of consideration of the extrinsic causes of abdominal pain. We should never forget the law that any irritation of a sensory nerve is referred to its end organ. This make clear the abdominal pains which are due to lesions of the spinal cord, or to diseases of the spine which affect the efferent nerves, such as caries, tumors, and arthritis deformans; also certain types of neuritis in the obdominal nerve distribution. In certain neurological cases abdominal pain may even be of central origin, explainable only by the hazy and dangerous mysticism of functional cerebral disorders.

Still other varieties of extrinsic abdominal pains are of the reflected variety, such as the epigastric crises of angina pectoris and the abdominal pains of basal pleurisy. These and other obscure conditions may present difficulties of diagnosis that are well-nigh insuperable and require the greatest amount of care in combining the results of the history and the examination.

The most familiar example of abdominal pain in which some spinal cord lesion is the underlying cause is the gastric pain of tabes. The differentiation is not difficult, since the gastric crises usually occur in the stage of the disorder when the symptoms are already sufficiently pronounced to enable a correct interpretation of the origin of the gastric symptoms. The most common cause for mistaking this condition for an intra-abdominal catastrophe is the failure to think of it. That the error is not uncommon, however, is shown by the series of cases of taobes reported by Nuzam from the Cook County Hospital, Chicago, in which he showed that approximately ten per cent. of about 1000 cases had been subject to abdominal operation for gastric or duodenal ulcer, gall-bladder disease, appendicitis, or other supposed lesions of the abdominal viscera. An example of pain referred to the lower right abdominal quadrant is that caused by acute epiphysitis of the upper end of the right femur; acure coxalgia of the right side; acute osteomyelitis of the right great trochanter can be the cause of pain being referred to the above region.

Fortunately nature has not drawn too many barriers across our trail, and in the vast majority of cases of abdominal pain the exciting cause is to be found in demonstrable disease of one or other of the occupants of the abdominal cavity. One must not too readily conclude that pain in the abdomen is of referred or reflected nature. It is possible for the tabetic to have abdominal disease, and I have operated upon the insane for appendicitis neglected until an abscess mass made the condition unmistakable.

That abdominal pain may be due also to a local manifestation of a general condition not amenable to surgical treatment is also true. The visceral crises of angioneurotic edema of purpura so well portrayed by Osler are examples, fortunately rare, of this type.

In general, therefore, while we may say that the alert clinician will keep a corner of his eye open for this extrinsic cause of abdominal pain, he will do well to center his full gaze upon the contents of the abdomen when pain dwells therein.

In general, abdominal pain is due chiefly to derangements of function resulting in tension, or to inflammation, or to both. The severe and rhythmic character of spasm or contraction of a hollow viscus is due to obstruction and represents the excess activity of the organ to break down the obstruction and the relaxation caused by fatigue resulting from the effort. We see this in its pure form most often in calculous disease of the genito-urinary and the biliary tract. Typically the pain in renal colic begins in the lumbar region and radiates forward and downward over the lower abdomen, and is referred to the genital organs. But how often do we see departures from the typical in the location, severity, and radiation of pain. The presence of stone in the right ureter in some of these cases not infrequently produces symptoms of appendicitis, a fact which should be borne in mind in the diagnosis of appendicitis. Ureteritis, stricture of the ureter, and pyelitis have been mistaken for appendicitis and the patient operated for the wrong thing; one of the watchwords is, always examine the urine. Typically gallstone colic, with its pain the right abdomen referred to the right shoulder and back, is familiar to all of us, but very often the pain is entirely located in the epigastrium, and it is then difficult to distinguish it from other diseases of the upper abdomen. In order to make the distinction it is necessary to be familiar with the clinical picture presented by other intra-abdominal affections, which we shall discuss presently.

Intestinal colic is another type of obstructive abdominal pain. This is too often attributed to some dietetic error, the most favorite diagnosis being ptomaine poisoning. The latter is in reality a rare condition, and, except in cases actually due to some tainted food, intense pain with vomiting and sweats and collapse will usually be found to be due to other causes, such as perforated viscus, pancreatitis, extrauterine pregnancy, abortion, or some septic condition of the abdomen. Severe intestinal colic is at times due to the presence of fecal masses, the removal of which soon relieves the condition.

Again, it may be due to the irritating effect of abnormal contents, causing inflammation and hyperperistalsis with resulting colicky pain. The most important decision to be made by the physician when confronted with the diagnosis of colic is to eliminate organic ogstruction. Regularly recurring cramps are significant of true obstruction. Hyperperistalsis from other causes rarely shows the regularity, the persitence, and the similarity of location that are characteristic of organic obstruction. Irregular intermittency of pain, with shifting position corresponding to the segment of the intestine which is undergoing the violent vermicular contraction, is indicative of tenteritis. As a rule obstructive pain is more severe than that of enteritis, though the latter may on occasion be equally severe. Naturally other symptoms, together with the history and the physical examination, enter largely into a diagnosis, but to-night I must merely point out that a painstaking development of the various characteristics of the pain in these various conditions is of greater value than is ordinarily appreciated, and, especially in doubtful cases, may be decisive.

There is one variety of colic about which we formerly heard much but fortunately nowadays but little is said. I refer to appendicular colic. I am willing to admit what is perfectly true, that the appendix suffers frequently from obstruction by concretion, strictures, kinks, torsions, and inflamatory swellings. That it occurs independently of inflammation of the appendix I am very skeptical. Appendicular colic is always appendicitis and it is a mistake to label it otherwise. If obstruction is the primary feature, at any rate inflammation treads upon its heels and in the vast majority of cases soon rules the scene. Appendicular colic, on the other hand, is a frequent result of appendicular inflammation. In this particular segment of the intestinal tract it is a mistake, therefore, to attempt to differentiate inflammation from obstruction. They are one and inseparable in the majority of cases, and the indications are the same.

As a general rule all agonizing abdominal pains belong to the surgeon. I trust my friends the internists( who would better be called externists, as they do all their work on the outside) will not think I am unduly grasping in making this assertion. We have fortunately outlived the age of sensory neuroses—''algias'' and other diagnoses, which were names only. We are approching the time when the surgeon will be called in every case of severe abdominal pain. To the excruciating group of abdominal diseases belong acute pancreatitis, mesenteric and mesocolic thrombosis, the ston colics, perforations of the gastrointestinal tract, extrauterine pregnancy, and twisted pedicles, all surgical conditions, and all conditions that may and often do call for prompt action to avert fatality. In striving for efficiency we should endeavor to cut down the latent period of diagnosis before treatment. The watchful waiting policy while pathology is making its onslaught cannot be other than disastrous. This can best be done by the promptest cooperation of the surgeon with the family physician.

One of the most common severe seizures of this group is without doubt that due to perforation of a gastric or duodenal ulcer. The pain is stab-like, intense, and is referred to the upper abdomen, which soon assumes the characteristic boardlike rigidity. Very often in these cases infection extending downward toward the right iliac fossa causes extreme tenderness in the appendiceal region and suggests acute perforating appendicitis and peritonitis—a diagnostic error of almost banal frequency and one which is not always avoidable even with a most carefully taken history. The operative mortality in perforating duodenal uscer in my experience is almost negligible—one death in 46 operations up to date. I do not hesitate to give all credit for this happy result to the prompt action on the part of an intelligent clientele of physicians.

The average case of acute appendicitis is readily recognized by most physicians, beginning, as it generally does, in the periumbilical region and soon localizing in the right iliac fossa. Yet the pain of this disease, both subjective and as elicited by examination, presents the most marked variations. Much depends upon the length of the appendix and the situation of its inflamed portion. For the sake of making an impression upon students I often say that if an appendix were to pass out of the abdomina cavity by way of either sacrosciatic foramen and continue its course through the popliteal space to the os calcis, and that person were to have appendicitis, he would have pain referred to his heel. Similarly a common cause of pelvic pain and left-sided tenderness is the pelvic appendix.

In many instances the seat of any lesion causing abdominal pain cannot be correctly surmised from the unaided description of the patient. In such cases we resort to the ordinary means of producing pain in the suspected location by pressure. A most useful expedient which I can recommend is to have the patient cough. In the presence of an acutely inflamed focus within the abdomen the impulse imparted to it by the sudden descent of the diaphragm will often point out to the patient and to the surgeon the exact location of the sore spot. This is particularly valuable in locating a diseased appendix before the early central pain has localized in the right iliac fossa. Per contra, it is true pain in the right iliac fossa in the female is oftentimes referable to some acute condition of the right ovary and of the Fallopian tubes. This is largely a matter of the position of the affected organ.

The pyloric orifice, the duodenum, the gall-bladder and biliary ducts, the head of the pancreas, and the hilum of the right kidney are all found within a very small area which can be covered by the palm of the hand upon the anterior abdominal wall, and the appendix is not distant. It is small wonder that these organs, accustomed as they are in a state of health to localization of sensation, should, when diseased, fail to speak an unmistakable language.

The pain of chronic gastric and duodenal ulcer, the most frequent site of which is near the pylorus, is epigastric and is sufficiently marked by its gnawing character, its periodicity and its relation to food intake, to make it unmistakable in typical instances. Gall-bladder disease causes pain in the right hypochondrium, radiating to the right. Involvement of the pancreas produces a low or central pain. The onset of pain in chronic appendicitis may suggest a lesion of almost any of the intra-abdominal organs mentioned, but it generally localizes in the appendiceal region, and the accompanying tenderness and rigidity of this region help to indicate the true nature of the trouble in the greater number of cases.

Intra-abdominal adhesions are often a convenient diagnosis with which to impress a patient whose abdominal pain does not suggest any definite lesion. I have elsewhere sounded a warning note, which I here repeat, against dangerous and difficult operation which have come into vogue for the relief of such supposed conditions, congenital or acquired. There is no doubt that pain of every degree may be caused by adhesions, but I again put the question, What is the normal arrangement of the abdominal viscera for a given individual? In reality we do not know, for people's viscera vary as much as does their physiogomy, though "in the image of God He created them all."

Pain of itself is not a clinical entity, and it is too often upon associated signs, such as local tenderness (referred pain never produces a tenderness at the referred area), spasm, vomiting, fever, chills, ets., and also on the history of the illness, that we must to a great extent rely in making our diagnosis, and finally on that sixth sense with which a favored few are endowed—intuition. The advances in abdominal surgery have contributed much to our knowledge of the immediate as well as the remote causes of pain. The early exploration has been responsible for bringing to the light of day the previously obscure causes of many varieties of abdominal pain.

Postoperative pain, real or fancied, has been one of the great bars to the general dissemination of many of the benefits of surgery. To avoid pain many have chanced the most horrible of deaths and lost. Our greatest regret is the pailess nature of malignant disease in its incipiency. But we must reckon with human nature and minimize to every degree consistent with safety the sufferings incident to surgery. In so doing I want to sound a warning note against the practice of narcotizing abdominal patients which has obtained recently in certain hight quarters, what I cannot refrain from calling pseudo-scientific support. I have passed through that struggle once and am now satisfied. If I want to know just where my patients are on the jojurney to recovery I do not want them drugged to stupefaction. I do not want to put their primitive vegetative functions at a disadvantage to pander to a weakness of the flesh. Morphia is truly God's own medicine, but only when used rightly and with discretion. The pains that occur after operation are in some instances like unto those that occur before operation, a warning. Even with the signal it may be difficult enough to sight the danger. Without it the patient may be upon the rocks and the surgeon taken unawares. The differentiation of abdominal pain occurring on the second or third day, or later, after operation is oftentimes puzzling. The pain of distention and obstruction is usually colicky and spasmodic, showing a definite relationship to peristalsis; there is generally neither tenderness nor rigidity; the degree of severity of the pain indicating whether it is being caused by distention or by obstruction. Postoperative peritonitis, on the other hand, produces muscular rigidity and tenderness, peristalsis is absent, the pain is constant and unremitting, the latter being, perhaps, the most marked distinction between peritonitis and posoperative pain of mechanical obstruction and distention.

We have thus far considered only the positive character of abdominal pain. There is also a negative side to the question—that is the absence of pain when it normally should be present. As in peritonitis it is that ominous silence of the belly wall that causes such mental distress to the surgeon, so also the sudden cessation of abdominal pain with or without the usual accompanying temperature and reduction in the leococyte count—portends a serious and grave state of affairs, indicating a ruptured or gangrenous appendix or peritoneal cavity, a condition not readily understood or recognized by the occasional operator or the tyro in surgery.

It is perhaps not out of place to say a word about painless abdominal conditions. I have on a number of occasions removed an intensely inflamed appendix as an incidental procedure during some abdominal operation, the patient being absolutely free from any pain referable to the condition prior to operation. Gastric and duodenal ulcers may exist for months and perhaps for years without any pain, or at least a minimal and trifling amount. Infection lurks at times in the gall-bladder from childhood to old age and only occasionally does suffering result therefrom. Yet these and other foci may serve as the laboratory in which toxins are manufactured and distributed to the body. They may be the portal of entry of many an obscure infection, and at bottom they may be responsible for a vast number of organic changes that masquerade under other names and are attributed to other causes. The degree of pain is not the index of treatment. It should be the aim of all of us to eradicate, not to palliate disease.

The recent extensions of the significance of infection we have no time to develop here, but we may note that pain does not always serve to point that which should be done. Yet pain is a faithful ally of both physician and surgeon, and thus we come back to extrolling its beneficient character. It is not a thing to be denied, a devil to be exorcised, but one of God's great gifts, worthy of our study, our admiration, and our praise.—The Therapeutic Gazette.

